# Considerations for mosquito microbiome research from the Mosquito Microbiome Consortium

**DOI:** 10.1186/s40168-020-00987-7

**Published:** 2021-02-01

**Authors:** Nsa Dada, Natapong Jupatanakul, Guillaume Minard, Sarah M. Short, Jewelna Akorli, Luis Martinez Villegas

**Affiliations:** 1 Faculty of Science and Technology, Norwegian University of Life Sciences, Ås, Norway; 2 Protein-Ligand Engineering and Molecular Biology Research Team, National Center for Genetic Engineering and Biotechnology, Khlong Neung, Thailand; 3 Univ Lyon, Université Claude Bernard Lyon 1, CNRS, INRAE, VetAgro Sup, UMR Ecologie Microbienne, F-69622 Villeurbanne, France; 4 Department of Entomology, The Ohio State University, Columbus, USA; 5 Department of Parasitology, Noguchi Memorial Institute for Medical Research, University of Ghana, Accra, Ghana; 6 Lund, Sweden

**Keywords:** Mosquito microbiome, Metabarcoding, Metagenomics, Metatranscriptomics, Recommendations for mosquito microbiome research, Microbiome data curation, Data quality, Reproducibility, Comparability, Microbial ecology

## Abstract

In the past decade, there has been increasing interest in mosquito microbiome research, leading to large amounts of data on different mosquito species, with various underlying physiological characteristics, and from diverse geographical locations. However, guidelines and standardized methods for conducting mosquito microbiome research are lacking. To streamline methods in mosquito microbiome research and optimize data quality, reproducibility, and comparability, as well as facilitate data curation in a centralized location, we are establishing the Mosquito Microbiome Consortium, a collaborative initiative for the advancement of mosquito microbiome research. Our overall goal is to collectively work on unraveling the role of the mosquito microbiome in mosquito biology, while critically evaluating its potential for mosquito-borne disease control. This perspective serves to introduce the consortium and invite broader participation. It highlights the issues we view as most pressing to the community and proposes guidelines for conducting mosquito microbiome research. We focus on four broad areas in this piece: (1) sampling/experimental design for field, semi-field, or laboratory studies; (2) metadata collection; (3) sample processing, sequencing, and use of appropriate controls; and (4) data handling and analysis. We finally summarize current challenges and highlight future directions in mosquito microbiome research. We hope that this piece will spark discussions around this area of disease vector biology, as well as encourage careful considerations in the design and implementation of mosquito microbiome research.

Video Abstract

**Supplementary Information:**

The online version contains supplementary material available at 10.1186/s40168-020-00987-7.

## Background

**Fig. 1:**
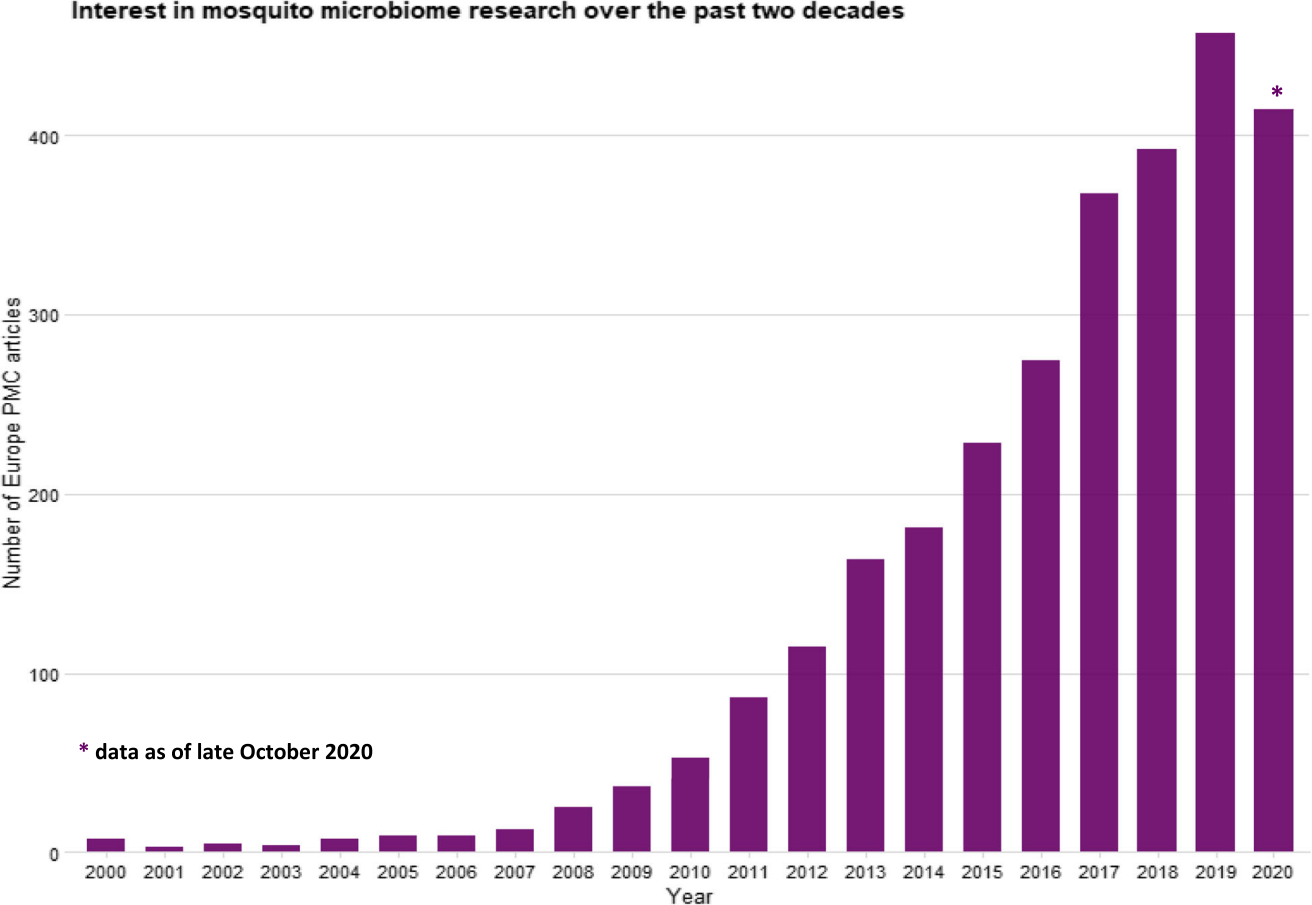
Barplot showing yearly number of publications in the Europe PMC archive from 2000 to date, using search query “mosquito microbiome.” The data show steadily increasing mosquito microbiome research publications over the past decade. Code for visualizing the most up to date trend is publicly available here: https://mosquito-microbiome.org/media/code-mosquito-microbiome-research-trends

The mosquito microbiome is critical for mosquito development, and it can have significant effects on vector competence, host immune system signaling, and longevity [1–5]. It has gained attention over the past decade for its influence on vector-borne pathogen transmission and as a potential avenue for vector-borne disease control. With this increasing interest, > 300 scientific publications on mosquito microbiome research per year can now be retrieved from scientific literature databases (Fig. 1). This increase in independent mosquito microbiome studies has led to large amounts of data on different mosquito species, with various underlying physiological characteristics, and from diverse geographical locations. It would be ideal for the data generated from these studies to be curated in a centralized location, as well as collected, analyzed, and stored in a way that would facilitate quick and easy access for various purposes including new hypothesis testing, development of novel research questions, and/or meta analyses. The creation of a centralized repository would be feasible if these data followed a set of guidelines to ensure optimum quality, reliability and comparability, and adequate records of metadata, as well as appropriate and rigorous data processing and analysis. These guidelines, which can be borrowed from existing knowledge and approaches of microbiome research of other living and non-living systems [6–8], should be tailored to and designed in collaboration with the international mosquito microbiome research community.

To facilitate creation of a curated data repository as well as collaboration and discussion among mosquito microbiome scientists, we are establishing the Mosquito Microbiome Consortium (www.mosquito-microbiome.org), a collaborative initiative for the advancement of mosquito microbiome research. With an emphasis on moving mosquito microbiome research from laboratory to field, our goal as a consortium is to collectively work on unraveling the role of the mosquito microbiome in mosquito biology, while critically evaluating its potential for mosquito-borne disease control. This initial piece serves to introduce the consortium and invite broader participation. It highlights the issues we view as most pressing to the community and suggests basic guidelines for conducting mosquito microbiome research. We hope that this will elicit more discussion from the scientific community that will feed into subsequent and more comprehensive recommendations for conducting reproducible mosquito microbiome research.

## Proposed guidelines for mosquito microbiome research

There are several comprehensive recommendations on best practices for microbiome studies [9, 10] that are very useful but not specific to the mosquito microbiome community. Gleaning from these recommendations, here we focus specifically on the mosquito microbiome and expound on specific components of the system. While there are many aspects of mosquito microbiome research that could be optimized and streamlined, we focus on four broad areas in this piece: (1) sampling/experimental design for field, semi-field, or laboratory studies; (2) metadata collection; (3) sample processing, sequencing, and use of appropriate controls; and (4) data handling, analysis, and deposition.

### Mosquito collections/experimental design for field, semi-field, or laboratory systems

Field sampling design will be highly variable depending on research questions, but should be formulated in advance of sample collection, following similar best practices as any mosquito ecology field study [11]. Factors to consider when designing field collections for a mosquito microbiome study may include number of sites, location of sites, distance between sites, and uniform sampling methods (e.g., timing, sample collection and handling across sites, and target mosquito tissue). Where immature mosquitoes and their breeding water are desired, it would be ideal to standardize the location in the water column across sampling sites from which the latter is collected. For adult mosquito sampling, methods that allow for the collection of live mosquitoes such as mechanical aspirators [12] or frequently checked adult traps, e.g., BG-Sentinel (Biogents, Regensburg, Germany) [13] or Mosquito Magnet® (Woodstream Corporation Lititz PA), should ideally be used.

While laboratory systems can be useful for investigating physiological interactions between mosquitoes and their microbiome, or investigating mosquito microbiome dynamics in a controlled setting, they are not always ideal for all research questions, largely because results obtained using laboratory populations cannot necessarily be extrapolated to field populations. Indeed, part of the microbial diversity might be lost or altered due to the influence of environmental factors in microorganism acquisition and maintenance in laboratory conditions [14, 15]. Approaches to isolate and experimentally re-introduce microbes from field populations into laboratory colonies can improve the applicability of laboratory studies [16, 17], and several factors that should be considered for optimizing the relevance of results of mosquito microbiome laboratory experiments for field mosquito populations have been discussed by Romoli and Gendrin [18]. Regardless, when designing a mosquito microbiome study, careful consideration should be given to whether the research questions can appropriately be addressed in a laboratory system and/or whether field or semi-field experiments should be conducted.

#### Individuals vs pools

An important factor to consider early in the design of any mosquito microbiome study is whether individual mosquito samples or pools of mosquito samples should be processed. This decision should primarily be informed by the overall goals of the study but can also be influenced by technical and financial constraints. Pooling samples limits the ability to investigate and account for variation between individuals and the ability to assess relationships between members of the microbial community within individual mosquitoes. There are however some benefits of pooling samples, such as allowing for a larger representative sample to be taken from a population or treatment, increasing biomass which could minimize the influence of contamination [19], and a reduction in the overall number of samples to be sequenced, thereby reducing sequencing costs. When individual mosquitoes are pooled for processing, biological replicates of pools should ideally be processed. Collecting and processing biological replicates can provide insights into variation within groups or populations, but analyzing individual samples (an adequate number to provide enough statistical power) where possible would be most ideal and informative [20]. This particular decision is of utmost relevance, as the microbiome data will have three inherent characteristics that are directly affected by the pooling scheme: dimensionality (pooling creates new groups), compositionality (revealed patterns and conclusions drawn from pools may not reflect individual composition), and zero-inflated counts (low frequency and/or undetected taxa may not be uniformly distributed across pools) [21]. Ultimately, and as tested by Rodríguez-Ruano et al. [20], the microbial diversity indices will significantly be influenced by the decision taken at this methodological crossroad.

#### Physiological characteristics

The physiological characteristics of mosquito samples selected for microbiome studies should be properly controlled in order to avoid introducing bias and/or confounding study outcomes, as the mosquito microbiome is known to affect or be affected by host physiological status [5]. Where possible and appropriate for the study goals, the use of mosquito samples with uniform physiological characteristics should be prioritized. In laboratory settings, this can be achieved by utilizing mosquito colonies that are reared under identical conditions. In field and/or semi-field settings however, two approaches can be considered: (i) rearing progeny from field-collected mosquitoes (ideally the first filial generation) under identical laboratory conditions or (ii) rearing field-collected immature mosquitoes in water from their breeding habitats—the study objectives would determine which of the two is appropriate. In studies where none of the above outlined approaches are feasible, for example, in cases where adult field-collected mosquitoes are required, (i) an effort should be made to determine the physiological characteristics of each sample, e.g., blood fed status, size/weight, and gravid status; (ii) individuals, rather than pools, should be processed if possible; (iii) if pools are desired, individuals with similar physiological characteristics should be pooled; and (iv) the potential impact of variable physiological properties on the microbiota should be adequately discussed when findings of such studies are presented.

#### Modification and manipulation of the mosquito microbiome

Some mosquito microbiome research questions may require controlled manipulation of the microbiota. Experimentally introducing microbes from field mosquitoes into laboratory colonies is a common approach for studying the effects of specific microbes on vector competence and physiology, e.g., [3, 16, 22]. Some research questions involving microbiota modification may call for the use of axenic or gnotobiotic mosquitoes. Antibiotics have been used in such studies until recently, but (i) antibiotics might directly affect the mosquito physiology or vector competence regardless of their impact on the microbiota [23], (ii) they do not fully clear the mosquito microbiota [24], and (iii) mosquitoes have been shown to harbor antibiotic-resistant microbes [25]. Recent developments in the creation of axenic and/or gnotobiotic mosquitoes [17, 26] offer promising solutions to these challenges and have revealed a critical role of the microbiota in larval development.

### Metadata

Regardless of whether samples are collected from the field or laboratory, a comprehensive record of sample characteristics, collection, handling methods, and experimental conditions should be maintained. This would not only benefit downstream data analysis and result interpretation, but would facilitate study repeatability and/or reproducibility, and allow for subsequent data reuse and reanalysis. Based on the FAIR (Findable, Accessible, Interoperable, and Reusable) guiding principles for scientific data management and stewardship [27], a minimum information standard for reporting arthropod abundance data, MIReAD [28], has been developed. MIReAD’s list and description of specific data fields that should be included in data collection sheets are relevant sample collection characteristics for mosquito microbiome studies, particularly those involving field collections. Other useful metadata standards are the Genomic Standard Consortium’s minimum specifications [29, 30]. Building on these existing standards, below we describe minimum metadata records to consider for mosquito microbiome laboratory, semi-field, and field studies. We also provide a checklist of these metadata records for quick reference (Table 1), along with a ready-to-use interactive and customizable template (freely available for download here: https://mosquito-microbiome.org/resources/mmc-white-paper/).

**Table 1:** Checklist of minimum metadata to be collected for mosquito microbiome samples and all controls (positive and negative) processed, along with examples following existing standards of recording and reporting arthropod and genomics data. A ready-to-use interactive and customizable template of these metadata records is freely available here: https://mosquito-microbiome.org/resources/mmc-white-paper/

Metadata fields	Example
***General***	
Study type	Field, semi-field, or laboratory
Sample name	*Anopheles gambiae* midgut, no template extraction/PCR control (negative control), ZymoBIOMICS microbial community standard or other known mock community (positive control), etc.
Sample ID	An_Gambiae_MG_01
Number per sample	Individual, pool of 3 individuals, etc.
Sample taxonomy	*Anopheles albimanus*
Developmental stage	Egg, larval instar, pupa, or adult
Sex	Male, female, or both pooled
Age^a^	3 days post adult eclosion, 2–5 days post eclosion etc.
Mating status	Virgin or mated/non-virgin
Gonotrophic status	Non-gravid, fully gravid, or half-gravid
Blood fed	Non-blood fed or blood fed
Type of food provided^b^	10% sucrose, human blood, 1:3 yeast and TetraMin, etc.
Tissue processed	Whole mosquito, midgut, ovaries, cuticle surface, etc.
Sample phenotype	Virus/parasite infection status, insecticide resistance status, etc.
Collection date	YYYY, YYYY-MM, or YYYY-MM-DD
Collection time	hh, hh:mm, or hh:mm:ss
Biomolecule processed	DNA, RNA, protein, metabolites, etc.
Biomolecule isolation method	QIAGEN blood and tissue, phenol-chloroform, etc.
Sequencing method	16S rRNA amplicon, whole (meta)genome, metatranscriptomic, etc.
Sequencing platform	Illumina, Oxford Nanopore, etc.
Sequencing platform model	MiSeq (Illumina), MinIon (Oxford Nanopore), etc.
Sample storage preservative	None, Ethanol, RNALater®, etc.
Sample storage temperature	− 20 °C, − 80 °C, etc.
Sample storage duration	None, 6 months, 3 years, etc.
***Specific to field studies***	
Collection country	Nigeria
Collection village or city	Iko Esai village
Location coordinates	00.000000, 00 00.0000, or 00° 00′ 00.0″ N 0° 00′ 00.0″ E
Climatic/environmental data	27 °C, 82% RH
Land cover	Savanna, urban and built up, tundra, etc. See Loveland et al. [31] for more examples
Collection method	Human landing catch, mechanical aspirator, gravid trap, or larval dipping
Collection bait	CO2, cattle
***Specific to laboratory studies***	
Name and location of laboratory (and/or facility)	The Short Lab, College of Food, Agricultural, and Environmental Sciences, The Ohio State University, Columbus, OH, USA
Strain	STECLA, KISUMU, ROCKEFELLER
Generation	F1, F6, F59, etc.
Maintenance temperature	27 ± 2 °C
Maintenance relative humidity	80 ± 10%
Light-dark cycle	10-h light; 14-h dark
***Specific to semi-field studies***	
Name and location of semi-field facility (if widely used/known)	MalariaSphere, Mbita Point Research & Training Center, International Centre of Insect Physiology and Ecology, Mbita, Kenya [32]
Type of semi-field structure	Glass house, mesh house, mesh cage, etc.
Dimensions of semi-field structure	00.00 × 00.00 m

^a^May not apply to field-derived mosquitoes

^b^Not applicable to field-derived mosquitoes

#### Metadata recommendations for both field and laboratory studies

##### Mosquito species

Ideally, records of mosquito species should not be limited to morphological identification because some species of medical importance are nested within complexes of cryptic species that cannot be distinguished from each other, e.g., *Aedes albopictus*, *Anopheles gambiae*, and *Culex pipiens* [33–35]. Thus, a molecular marker should be employed to adequately identify samples—the mitochondrial cytochrome c oxidase subunit 1 (COI) gene is the most referenced molecular marker for distinguishing between eukaryotic taxa, and the Malaria Research and Reference Reagent Resource Center (MR4) provides detailed protocols for distinguishing mosquito species using the COI gene [36]. Alternatively, the internal transcribed spacer (ITS) can also be used for species identification [37].

##### Mosquito developmental stage/age and sex

Mosquitoes develop through four stages—egg, larva, pupa, and adult, and the pupal and adult stages are sexually dimorphic and distinguishable. Records should include (a) mosquito developmental stage, as well as age for adults where possible and relevant, and (b) sex of pupae/adults. This is particularly important because the mosquito microbiome is known to be affected by these physiological characteristics or vice versa [38, 39]. It may be difficult to determine the age of field-collected adult mosquitoes, but with new technological applications such as machine learning and infrared spectrophotometry, their age can be estimated [40]. Also, with improved annotation of sex loci in genomes of some mosquito genera [41, 42], it may be possible to distinguish between male and female eggs and larvae in the future via molecular techniques. Mosquito larvae develop through four larval instar stages (L1–L4). If larvae are used, the instar stage should be recorded where it is possible to distinguish instars. In species or cases (e.g., following preservation) where this is not possible, larval instars can be grouped as early (small, light colored, typically L1–L2) or late (larger, darker, and typically L3–L4).

##### Mating, parity, feeding, and gonotrophic status

Mosquito adult life history characteristics including mating, parity, feeding, and gonotrophic status may influence or be affected by host microbiome [43, 44]. It is thus important to record these physiological characteristics for samples when possible. These factors can easily be controlled and subsequently recorded for laboratory strains and/or progenies of field-collected mosquitoes that are reared under laboratory/controlled conditions. For example, male and female mosquitoes can be separated at the pupal stage to obtain virgin adults if mating and parity need to be controlled, type of food and length of time post feeding can be controlled, and gonotrophic cycle can be observed post blood-feeding. For field-collected mosquitoes, while the gonotrophic status can be determined by examining the abdomens of collected female mosquitoes, other physiological characteristics may be challenging to obtain. Determining these other physiological characteristics may require the removal and microscopic examination of specific tissues, e.g., female spermatheca and ovaries for mating and parity status [45, 46] or a newer non-invasive procedure as described for *Ae. aegypti* [47], and the use of metabolomic or metabarcoding approaches to determine food sources [48–51]. The former may be feasible where these specific tissues are targeted for the microbiome study, and the latter, where it is possible to extend laboratory processes to incorporate metabarcoding/metabolomics.

##### Tissue/organ studied

To date, mosquito microbiome studies have focused on entire individuals [52, 53], the alimentary canal [15, 54–59], salivary glands [56, 60], and/or reproductive tissues [24, 56], with a recent study extending microbially characterized mosquito tissues to the cuticle surface [61, 62]. Since many members of the mosquito microbiome show tissue tropism [39, 61], it is important to report what specific tissues are being analyzed, where applicable, or that whole samples where processed.

##### Storage and handling conditions

Sample storage conditions have been shown to introduce variability in microbiome studies [63]. Thus, the preservation method used (e.g., buffer, preservative, temperature) along with storage duration, including any freeze-thaw cycles and/or transportation chains (e.g., transportation on ice/dry ice/ambient temperature), should be recorded for each study. Study objectives and available resources would influence the choice of sample storage and handling, and previous research on how different methods affect insect microbiomes, e.g., [20, 64, 65], could inform these decisions. Irrespective of chosen methods, each study should ideally use a single storage method to prevent the introduction of batch effects. Where this is not possible, for example when working with historical samples, batch effect should be accounted for during data analysis and its effect subsequently discussed.

##### Sample processing, sequencing, and controls

Each sample processing step, the type of sequencing approach employed, along with the type of controls included in each step should be recorded. The Genomic Standard Consortium’s minimum specifications [29, 30] can be used as a baseline.

#### Metadata recommendations that are specific to field studies

##### Location

It is essential to record the origin and/or collection site of mosquito samples, as the mosquito microbiome is known to vary spatially [53, 66, 67]. These records would facilitate study replication, reproducibility, and/or spatiotemporal analysis. In addition to the location name, the GPS coordinates of mosquito collection sites should be recorded with the most precise geodetic system. The best choice is often the local system which can subsequently be converted into international standards for example using the World Geodetic System. It should be noted that while the collection or breeding sites of immature mosquitoes represent sample origin, this might not be the case for adult mosquitoes, as some species can fly up to 50 km from their breeding sites [68]. Thus, for field-collected adults, sampling location, rather than sample origin, should be indicated in the metadata records.

##### Climatic and environmental data

As with location, the mosquito microbiome shows seasonal [69, 70] and environmental [71] variation. Thus, where possible and applicable, climatic and environmental data such as temperature, humidity, atmospheric pressure, land cover, and proximity to human dwellings should be recorded. The environmental type (e.g., breeding water or atmospheric) and geographic scale (e.g., microscale data collected with a data logger or macroscale data collected from a meteorological station) to which the data applies should also be indicated, along with the time scale over which the data apply. For larval breeding sites, water quality indicators can also be recorded, including pH, total dissolved solids, and salinity, in addition to land cover information of the surrounding area. Where climatic data or land cover data are collected from national or international databases [72], these should be indicated.

##### Collection method

The type of collection method or tool used for sample collection can influence the type and characteristics of mosquitoes collected [73, 74]. Thus, methods that are appropriate for the target species, life stage, and physiological characteristics should be employed and recorded. Each study should ideally employ a single sample collection method to avoid introducing bias. Some points to consider when collecting samples are described in the “Mosquito collections/experimental design for field, semi-field, or laboratory systems” section above.

#### Metadata recommendations that are specific to laboratory studies

##### Mosquito strains

Although environmental factors appear to be a major driver of mosquito microbiome composition, some studies also show the contribution of host genetic characteristics [57, 67, 75–77]. Different mosquito genotypes may have different microbial compositions due to genetic variation in host physiological or immunological traits, or the vertical transmission of symbionts [44, 77–79]. Thus, the mosquito strains used should be reported, particularly if they are well documented and broadly used. If the strains are more specific to the laboratory or the experiment, their origin and the process used to generate and maintain them should be reported.

##### Filial generation and maintenance of mosquito strains

During laboratory experiments, mosquito strains are often maintained over several generations. Vertically transmitted symbionts could be maintained over multiple generations, but environmentally or horizontally acquired symbionts are likely to be lost [62]. Reporting the filial generation of mosquito strains used would inform any variation that occurs in the mosquito microbiome. In addition to the number of generations, it would be valuable to report conditions and methods used in maintaining the mosquito strains. Examples of such conditions include: egg dessication between generations, synchronized egg hatching (using  a vacuum for example), egg disinfection (for example with bleach solution), type of water used for breeding, larval diet recipe, and source of blood for adult feeding. These factors would inform any shifts in microbial community composition across generations.

#### Metadata recommendations that are specific to semi-field studies

Semi-field characteristics play an important role in mosquito physiology and behavior [80, 81], both of which are known to affect or be affected by the mosquito microbiome [2, 4]. Thus, in addition to metadata collected for field or laboratory studies (depending on the mosquito source), semi-field characteristics should be recorded. These should include the description and dimensions of the semi-field structure or the name, along with a reference of the facility if it is widely known and used (e.g., the semi-field system at Ifakara Health Institute, Kilombero, Tanzania [81], or MalariaSphere at the International Centre of Insect Physiology and Ecology, Mbita, Kenya [32]).

### Sample processing, sequencing, and use of appropriate controls

Isolating and processing the microbiome from biological samples involve several steps including sample handling, nucleic acid/peptide/metabolite isolation, purification, amplification (where necessary), identification, and sequencing. All these steps can be accomplished in-house using commercially available kits, in-house generated methods, or a combination of the two. Alternatively, all or some of the steps (commonly the sequencing step as the machines are usually expensive to own) can be outsourced to commercially available facilities. While the method of choice would depend on research questions and available resources, each study should ideally employ the same methods for all samples to avoid the introduction of batch effects. Samples should also be handled and processed to avoid the introduction of extraneous microbes. Below, we describe sample processing steps in detail, highlighting points to consider for optimal quality and comparability.

#### Sample handling and preservation

After collection, samples should be handled to minimize alterations to the microbial communities, and/or nucleic acid degradation prior to isolation. This requires some planning ahead. In some cases, immediate freezing of samples at − 20 °C or lower using a freezer, dry ice, or liquid nitrogen may be possible. Alternatively, whole samples or dissected target tissues can immediately be homogenized and stored in lysis buffer or commercial nucleic acid preservative. In the field where immediate storage may not be possible, live samples can be collected and transported to the laboratory or field station for proper storage—immature mosquitoes should ideally be transported in their breeding water. Sample handling and preservation methods may differ depending on target biological macromolecule. For example, methods that are adequate for preserving the DNA and amino acid contents of biological samples may not be adequate to preserve RNA. Thus, sample handling and preservation should be carefully considered, and decided upon prior to sample collection.

#### Isolation and purification of microbial genetic material

The methods used for isolating and purifying genetic material would largely be determined by the study objective, genetic material of interest, and intended sequencing approach. These factors thus need to be carefully considered in selecting appropriate methods. Nucleic acids and proteins are largely targeted for microbiome studies, and currently, three main sequencing methods are widely employed: metabarcoding (targeting 16S rRNA/18S rRNA/ITS genes), metagenomic, and metatranscriptomic sequencing. The isolation and purification methods would differ by type of nucleic acid, as well as targeted genetic material. For instance, in metabarcoding where the universal bacterial and archaeal 16S rRNA gene or eukaryotic 18S rRNA gene and ITS region are selectively targeted with specific primers, contamination of mosquito genetic material may not be an issue. However, for metagenomic as well as metatranscriptomic sequencing, the outputs could be dominated by the genetic material of the mosquito host whose genomes—ranging from 0.098 to 1.8 GB [82]—can be larger by several orders of magnitude compared to that of mosquito-associated microbes. In this case, the nucleic acid isolation and purification step could be (depending on study objectives) approached such that the mosquito cells/tissues are selectively digested, and/or microbial cells/DNA are enriched prior to nucleic acid isolation and purification [83–87]. The selective digestion of eukaryotic host cells/tissues is especially possible for studies where prokaryotic microbial communities are the focus, as it may have minimal impact on the prokaryotic cells. A similar approach of selectively targeting prokaryotic RNA [88] can be employed for metatranscriptomic studies.

Both in-house protocols and commercially available nucleic acid isolation kits have been used in microbiome studies [89]. However, to reduce contamination and allow for reproducibility, commercially available kits are preferable. Irrespective of preference, the protocol selected should be appropriate for isolating the genetic material of targeted microbial components, as nucleic acid isolation and purification protocols vary in their efficiency depending on template biological material [90]. In addition, one nucleic acid isolation and purification method should ideally be employed for processing all samples within a study, as results of microbiome composition analyses have been shown to vary by nucleic acid isolation methods [91–93].

#### Sequencing (‘omics) approaches

As earlier mentioned, metabarcoding, metagenomic, and metatranscriptomic sequencing, in addition to metaproteomics, are employed in microbiome studies. While metabarcoding involves sequencing of amplified gene targets to describe the composition of microbial communities, the other approaches provide the same insights, along with functional characterization of the microbial communities, but without the biases of targeted gene amplification. Well-curated and regularly updated reference databases based on commonly targeted microbial genetic markers (e.g., the SILVA ribosomal RNA gene database project [94]) allow for high taxonomic resolution with metabarcoding. In contrast, it can be challenging to accurately infer taxonomic origin using other ‘omics approaches, such as shotgun metagenomics and metatranscriptomics due to limited well-curated databases. However, these ‘omics approaches provide broader and more informative outcomes including functional catalogs, and expression profiles, in addition to taxonomic annotation of the community members. The choice of sequencing approach for mosquito microbiome studies should thus be carefully selected based on research objectives as well as the strengths and weaknesses of the different approaches [9, 95]. As with other microbiome studies, mosquito microbiome studies have predominantly focused on 16S rRNA amplicon gene sequencing. This approach and its associated data analysis tools are thus widespread and easily accessible [96–102]. In other disciplines, there have been increasing shifts from descriptions of microbial community composition to using other ‘omics tools to infer and understand the functions of microbial communities [103–105]. However, the mosquito microbiome research community is only just beginning to utilize these other ‘omics approaches to understand the functions of the mosquito microbiome and its role in vectorial capacity [83, 106–108].

#### Appropriate controls

In conducting any microbiome study, care must be taken to avoid the introduction of any extraneous microbes, as this can invalidate study outcomes. This can be accomplished by working under sterile conditions, and processing blank controls (no sample or template) along with samples at each sample processing step, to capture potential contamination by extraneous microbes [19, 109–111]. Each mosquito microbiome study should include blank controls at each of the following steps where applicable: sample collection (including surface sterilization and dissection where applicable), nucleic acid isolation and purification, PCR amplification, and library preparation. Controls from a previous step should be processed as a sample in subsequent steps in addition to new controls for that step, and ideally controls should be used for each batch of samples processed where they are not all processed at the same time/by the same person (batch processing should be avoided as much as possible, unless processing and/or sequencing samples in multiple batches are necessary to account for or estimate a specific parameter identified in advance). Where feasible, and especially important for high-throughput sample processing, positive controls (i.e., mock microbial community or template) should be included to optimize and validate each of these steps, as well as to capture any extraneous and cross-contamination. It is important to note that the blanks may not pass pre-sequencing quality control procedures due to absence of or low microbial load; however, they should be sequenced irrespective of whether they pass the pre-sequencing quality control or not. Several studies have shown that blanks that do not present bands following PCR and/or fail the pre-sequencing quality control step result in sequences [83, 112] that can subsequently be excluded from samples in downstream analysis [57, 113]. All steps taken to minimize contamination, along with any contamination captured, should be reported in published studies.

#### Biological and technical replicates

The use of biological replicates has been highlighted in the “Mosquito collections/experimental design for field, semi-field, or laboratory systems” section (Individuals vs pools), but bears repeating as results without biological replicates are inconclusive due to failure to account for heterogeneity and/or variability in the samples or population. While technical replicates may be optional, depending on the research question [114], biological replicates are not [115]. The number of replicates would depend on the research questions, approach used, and, in some cases, available resources. If in doubt, a statistician or bioinformatician (specializing in microbiome studies) should be consulted during the study design.

### Data processing, analysis, and deposition

Irrespective of system, the same overarching principles of processing, analyzing, and depositing microbiome data apply. Several review articles, including those by Knight et al. [9], Hongzhe [116], and Morgan and Huttenhower [117], discuss available methods for analyzing microbiome data. These reviews categorize analysis by ‘omics approach and provide strengths and limitations of the methods discussed. There is a plethora of tools available for analyzing microbiome data for example phyloseq [102], mothur [101], QIIME2 [118], Anvi’o [119], and MEGAN6 [120, 121], most of which require some knowledge of programming, and this could pose a challenge for mosquito microbiome research (see below). Web-based and/or graphical user interface (GUI) versions of some of these tools, which require little or no programming knowledge, are available or are in different stages of development. For example, the Galaxy platform [122] offers a variety of tools for analyzing microbiome data. For an example guide to statistical analysis in microbial ecology, see Buttigieg and Ramette [123]. Like metadata, a good record of each data processing and analysis step, along with any codes/scripts used, should be kept. These along with the metadata and sequences (including those of controls) should be made publicly available upon study publication. Different public repositories, along with instructions for data deposition, are available; the most popular ones include the NCBI Sequence Read Archive [124], EMBL-EBI European Nucleotide Archive [125], and the NIG DNA Data Bank of Japan [126]. If studies are properly conducted, “negative” or “unexpected” results should not preclude publication of the data, as several journals now specialize in publishing genomic data.

## Future directions and challenges in mosquito microbiome research

Studies showing how the mosquito microbiome influences mosquito physiological characteristics, including vectorial capacity [3, 18], life history traits [16, 17], blood meal digestion, fecundity [127], and insecticide resistance [61, 83], highlight different perspectives of mosquito-microbe interactions that are being further explored as new avenues for mosquito and mosquito-borne disease control. Standardized methods for conducting mosquito microbiome research, along with a well-curated repository of mosquito microbiome data, would expand the application of mosquito microbiome studies even further. Below are some future directions, along with some of the challenges of mosquito microbiome research:

i. *Mosquito microbiota as indicators of host life history*. As indicated by studies on habitat/location [53, 128–130], food source [43], age [131], infection status [18], and insecticide resistance [61, 83], more comprehensive research on the mosquito microbiome, particularly covering wider geographical locations and more mosquito species, could potentially be used to determine the ecological and physiological life histories of field-collected mosquitoes. It is relevant to highlight current research on how environmental change and human activities are influencing epidemiologically relevant mosquito behaviors [132–134]. So far, one published study on the links between the larval ecology of malaria vectors and malaria transmission has incorporated larval microbiota [71]. The extent to which the mosquito microbiome reflects or is involved in these ecological and epidemiological dynamics requires more attention.
ii. *Microbiome immune priming of mosquitoes against pathogens*. Although studies have demonstrated that the mosquito microbiome influences vector competence for malaria parasites and dengue viruses [22, 135–138], the direct impact of collective microbiome-induced immune activation (microbiome immune priming) on vector competence is much less described. Immune priming, driven by specific microbes, has however been demonstrated in mosquitoes [137, 139–146]. With this existing knowledge, and those of specific mosquito immune responses that control pathogen infection, particularly against malaria parasites [137], future research could extend to collective microbiome immune priming and its effect on vector competence. This knowledge could be further exploited for disrupting pathogen infections in mosquito populations, as recently shown in dengue control using *Wolbachia* strain wAlbB [138].
iii. *Mosquito microbiome as vehicles of pathogen transmission disruption*. Natural mosquito symbionts are readily able to colonize and inhabit the mosquito host. They can colonize specific host tissues [56], and can be vertically transmitted, including those that affect the fate of pathogens within mosquitoes, e.g., *Serratia marcescens* [147], making them suitable candidates for paratransgenesis [148]. Rapid advances in genetic engineering technologies [149, 150] make paratransgenesis a promising tool for disrupting the development of pathogens within the mosquito host, and significant advances have already been made for malaria parasite infections [151, 152]. More work targeting additional pathogens and particularly concentrating on field mosquito populations is required in this area.
iv. *Microbiome-derived metabolites for mosquito-borne disease control*. The diverse mosquito microbiome provides a microbial repertoire that can be explored for metabolites or enzymes with anti-pathogen and/or mosquitocidal activity [153], with recent studies identifying antimalarial [154], antibiotic [155], and insecticidal [156, 157] compounds from the mosquito microbiota. As seen in microbiome research in other systems such as plants [158] and humans [159], an advancement in this area would include connecting mosquito microbe-derived metabolites to their biosynthetic gene clusters as exemplified by Ganley et al. [160]. Expanding research efforts towards harnessing the strengths of bioinformatics in combination with functional ‘omics tools such as metatranscriptomics, metaproteomics, and metabolomics would drive this area of mosquito microbiome research forward. In addition, this area of research would also benefit from a well-curated mosquito microbiome data repository that also includes data on biosynthetic gene clusters from mosquito-associated microbes.
v. *Mosquito microbiome for mosquito population suppression*. Mosquito-derived microbes have long been considered for mosquito population suppression [161, 162], and advances in this area of research have resulted in field applications of mosquito endosymbionts for mosquito population suppression. So far, *Wolbachia* spp. have been employed [163–165] with varying outcomes. Briefly, the introduction of exogenous (from other mosquito/insect species) *Wolbachia* endosymbionts into mosquitoes decreases male mosquitoes’ ability to reproduce with natural females, a method called “Incompatible Insect Technique” (see Lees et al. [166] for a review). Another widely used mosquitocidal microorganism is the entomopathogenic bacteria *Bacillus thuringiensis* subspecies *israelensis.* When sporulating, this gram-positive bacterium produces both deltaendotoxins (*cry*) and hemolytic factors (*cyt*) with high larvicidal activity against *Aedes*, *Culex*, and *Anopheles* mosquito species [167]. Further work to dissect interactions between mosquito microbiome and mosquito life history traits could uncover new natural mosquito symbiont candidates for mosquito population suppression.
vi. *Updated ‘omics approaches and expansion beyond the prokaryotic component of the microbiome*. Mosquito microbiome research would benefit from expansion to non-prokaryotic components, including non-pathogenic and insect specific viruses, phages, fungi, and other eukaryotic microbes, that have largely been neglected. Growing evidence demonstrates that these non-prokaryotic microbes may play important roles in mosquito biology and vector competence [168–170]. Mosquito microbiome studies using different ‘omics approaches and those focusing on neglected microbes are still evolving, with currently limited available reference genomes for mosquito-associated microbes [160]. Future efforts should be directed towards augmenting the number of available reference genomes for mosquito-associated microbes and/or identifying currently available orthologs from other systems. This would subsequently facilitate the use of ‘omics tools other than metabarcoding for deeper insights into the role of the mosquito microbiome, and better resolution of taxonomic annotation—as the accuracy of taxonomic delineation using currently available metabarcoding tools (e.g., 16S rRNA gene) is limited [171]. There also needs to be a shift from our current practice of merely describing mosquito microbiome composition to incorporating how this composition affects different host characteristics; this is slowly changing with increasing access to molecular tools.
vii. *Better understanding of the complex mosquito microbiota interactions and networks*. Mosquito microbiome research is limited by our scant understanding of the complexity of the mosquito microbiome [57, 172–174], the complex interactions between the microbes, as well as interactions between the microbial network [175], the mosquito host and the pathogens which may infect them. Microbes reside in mosquito tissues as a community [56], and may need to be present as a community to affect mosquito physiology. Studies that have investigated the relationship between the mosquito microbiome and some aspects of host physiology uncover relationships that are challenging to parse, as it is unclear whether the microbiome influences a physiological outcome, vice versa or both [2, 83]. This offers opportunities for future work to unravel these complex interactions.
viii. *Unraveling the determinants of mosquito microbiome composition*. Some studies suggest that the environment may be a major determinant of mosquito microbiome composition [176, 177], while others suggest a highly dynamic composition that is controlled by several factors [66, 178] including host species [52, 71, 76]. In addition, multiple studies have documented inter-individual variations among field-collected mosquitoes [52, 57, 172, 174]. And while some studies have also demonstrated this variation in laboratory-reared mosquitoes [75, 77, 179], others have shown little to no variation [54]. Taken together, this presents several hypotheses pointing towards either a niche-, function-, evolutionary-, or stochastically determined microbiome [70, 180–183]. A large role for stochasticity could pose problems for mosquito microbiome manipulations in the field or the use of the microbiome as a predictive variable for modeling vector-borne disease transmission. High priority should therefore be placed on improving our understanding of the determinants of mosquito microbiome composition.
ix. *Biological validation of mosquito microbiome findings*. Another challenge in mosquito microbiome research is limited biological validation of results and inferences. This largely requires culturable bacteria, and large portions of the mosquito microbiome are non-culturable [83, 172, 184, 185]. Employing synthetic biology to transfer genes from non-culturable microbes into culturable symbionts could be one way to address this limitation. While synthetic biology may offer a way around this limitation, data on the role of the microbiome in host physiology—particularly pathogen infection—is lacking for field mosquito populations (but see [186]). This could be in part due to the challenge of finding infected mosquitoes in the field, but with a concerted effort, along with streamlined methods, this could be rectified. Going forward as a community, especially as our focus shifts towards harnessing the mosquito microbiome for mosquito and mosquito-borne disease control, we need to better establish and validate, in field populations, the role of the microbiome in mosquito physiology and vectorial capacity.
x. *Access to improved infrastructure and capacity*. While rapid advances in, and increasing access to, molecular technologies have led to an increase in mosquito microbiome studies, analysis of the resulting data remains a major challenge, as specialized bioinformatics skills along with expensive computing resources are required. This is particularly true in parts of the world where mosquito-borne diseases are endemic. In these areas, access to advanced molecular technologies is also limited. More funding to support mosquito microbiome research, particularly for improved laboratory facilities, long-term establishment of computing resources, and bioinformatics training (in endemic countries especially), would be practical for addressing this challenge. Challenges that are inherent to microbiome sample processing and data analysis have extensively been discussed [9, 187]; these also apply to mosquito microbiome research.

## Summary and conclusions

Guidelines and standardized methods are needed for reproducibility, replicability, and comparability in mosquito microbiome research. In this perspective, we address issues that we find most pressing in mosquito microbiome research and propose some guidelines to allow for more streamlined research.

Some good practices to consider for mosquito microbiome studies include adequate design, appropriate sample collection and processing methods, inclusion of appropriate controls and replicates, and the use of up-to-date data analysis tools. Open data sharing principles must be adhered to. This would include appropriate deposition of collected data, including raw files, data matrices, metadata, and analysis scripts. This would particularly be useful for creating curated repositories that could be incorporated into existing microbiome or mosquito/insect genome repositories such as MicrobiomeDB [188], VectorBase [189], FlyBase [190], InsectBase [191], or i5kworkspace@NAL [192].

As a community, we would benefit from more sample/data sharing and collaborations, and with streamlined methods, our work would be more reproducible, replicable, and comparable. This perspective serves as a starting point for streamlining mosquito microbiome research methods, and a call for researchers with interests in any aspect of this evolving vector biology niche to share their thoughts on appropriate methods for conducting mosquito microbiome research. Harmonizing research methods and creating a well-curated repository of mosquito microbiome data will provide a valuable resource that can be mined for new microbes/microbial agents for mosquito and mosquito-borne disease control. Our near-term goal is to make this mosquito microbiome data repository available and accessible to all.

## Data Availability

More information about the Mosquito Microbiome Consortium can be found at www.mosquito-microbiome.org. The code used to generate the mosquito microbiome research trend, along with the metadata checklist and ready-to-use customizable template, has been made publicly available and can be obtained from www.mosquito-microbiome.org/resources/mmc-white-paper/.
